# Model of collective detachment in high-grade serous ovarian cancer demonstrates that tumor spheroids produce ECM to support metastatic processes

**DOI:** 10.1063/5.0132254

**Published:** 2023-02-28

**Authors:** Hannah M. Micek, Lauren Rosenstock, Yicheng Ma, Caitlin Hielsberg, Lauren Montemorano, Metti K. Gari, Suzanne M. Ponik, Pamela K. Kreeger

**Affiliations:** 1 Department of Biomedical Engineering, University of Wisconsin-Madison, Madison, Wisconsin 53705, USA; 2 Department of Obstetrics and Gynecology, University of Wisconsin School of Medicine and Public Health, Madison, Wisconsin 53705, USA; 3 Molecular and Cellular Pharmacology Training Program, University of Wisconsin School of Medicine and Public Health, Madison, Wisconsin 53705, USA; 4 Department of Cell and Regenerative Biology, University of Wisconsin School of Medicine and Public Health, Madison, Wisconsin 53705, USA; 5 University of Wisconsin Carbone Cancer Center, University of Wisconsin School of Medicine and Public Health, Madison, Wisconsin 53705, USA

## Abstract

High-grade serous ovarian cancer (HGSOC) metastasizes through transcoelomic spread, with both single cells and spheroids of tumor cells observed in patient ascites. These spheroids may form through single cells that detach and aggregate (Sph-SC) or through collective detachment (Sph-CD). We developed an *in vitro* model to generate and separate Sph-SC from Sph-CD to enable study of Sph-CD in disease progression. *In vitro*-generated Sph-CD and spheroids isolated from ascites were similar in size (mean diameter 51 vs 55 *μ*m, p > 0.05) and incorporated multiple ECM proteins. Using the *in vitro* model, nascent protein labeling, and qRT-PCR, we determined that ECM was produced after detachment. As fibronectin plays a key role in many cell adhesion events, we confirmed that inhibiting RGD-based adhesion or fibronectin assembly reduced Sph-CD-mesothelial adhesion strength under shear stress. Our model will enable future studies to determine factors that favor formation of Sph-CD, as well as allow investigators to manipulate Sph-CD to better study their effects on HGSOC progression.

## INTRODUCTION

The five-year survival rate for ovarian cancer patients remains under 50%, with little improvement in outcomes over the past twenty years.^1^ Most ovarian cancers are diagnosed at advanced stages after abdominal or distant metastasis has occurred, where survival drops to 30.8%. High grade serous ovarian cancer (HGSOC) is the most common histologic subtype of ovarian cancer and metastasizes through transcoelomic spread, where single cells or spheroids of cells detach from the tumor and travel through the peritoneal cavity in peritoneal fluid/ascites. These cells then seed onto peritoneal organs, including the omentum, mesentery, and peritoneal lining. Spheroids have been shown to have increased resistance to anoikis and chemotherapeutics compared to single cells,^2–4^ suggesting they would have an advantage during metastasis. Therefore, understanding mechanisms that regulate spheroid formation, transport, and reattachment could identify new therapeutic targets to slow metastasis in HGSOC.

Spheroids can form through single cells that detach from tumors and aggregate in the peritoneal fluid/ascites (Sph-SC) or through collective detachment (Sph-CD). It was previously assumed that most spheroids in HGSOC were Sph-SC, but studies within the last decade have demonstrated that formation of Sph-CD occurs in HGSOC cells *in vitro* and *in vivo.*^5–7^ For example, orthotopic injection of mCherry or GFP-tagged OV90 cells into contralateral ovaries in mice generated largely single-color spheroids in ascites, suggesting spheroids formed through collective detachment or aggregated very quickly after detaching.^6^ Several groups have noted that cells in culture can produce 3D “buds” that extrude from a monolayer as a collective process.^5,7^ However, analysis of Sph-CD has been limited, in part, because of limitations with current model systems. Spheroids isolated from *in vivo* or the media of cells in culture are a mixed pool of Sph-CD and Sph-SC, and observation of Sph-CD formation by imaging methods is low throughput. Methods to generate spheroids *in vitro* rely on culturing cells at high densities in non-adherent conditions to encourage aggregation (e.g*.,* hanging drop, Aggrewell). Though these methods are facile approaches to generate large numbers of viable spheroids and mimic aspects of Sph-SC, these approaches do not recapitulate collective detachment to answer questions about this process or the resulting Sph-CD. Therefore, there is a need to generate an *in vitro* model where spheroids can form through either collective detachment or single cell aggregation and be easily separated for further characterization.

The ability to isolate spheroids formed from either single cell aggregation or collective detachment would enable the study of variables that influence spheroid survival and seeding of metastatic sites, such as the extracellular matrix (ECM). In this study, we developed an *in vitro* model to generate spheroids that were enriched for Sph-CD. We characterized the size and ECM molecule expression of Sph-CD generated in this system and compared these metrics to spheroids isolated from patient ascites, and then examined mechanisms that influence seeding of Sph-CD in the metastatic site.

## RESULTS

### Characterization of spheroids isolated from patient ascites

**FIG. 1.**
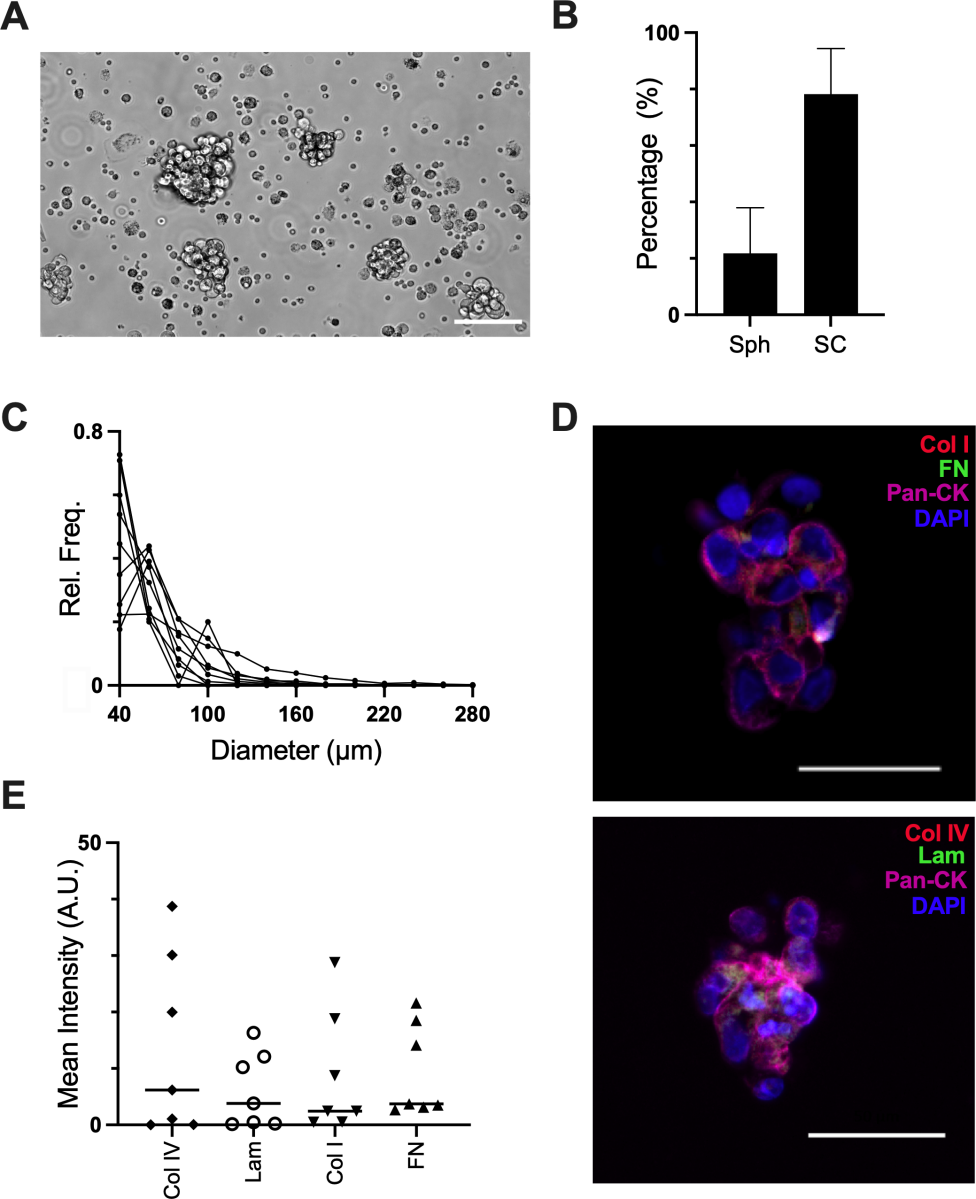
Characterization of spheroids found in ascites from patients with Stage III/IV HGSOC. (a) Phase images of spheroids isolated from ascites. Scale bar = 100 *μ*m. (b) Percentage of objects in ascites that were spheroids vs single cells. (c) Histogram of the size distribution of spheroids isolated from ascites, each line is an individual patient [n = 7 patients for (b) and (c)]. (d) Representative images of immunohistochemistry of spheroids from ascites. Top: collagen I (red), fibronectin (green), pan-cytokeratin (pink), nuclei (blue). Bottom: collagen IV (red), laminin (green), pan-cytokeratin (pink), and nuclei (blue). Scale bar = 50 *μ*m. (e) Quantification of ECM in spheroids, n = 7 patients, with each data point representing the average signal of 5–25 spheroids per patient.

We first sought to characterize the size, cell type, and ECM expression of spheroids from ascites in HGSOC patients. Spheroids and single cells from ascites fluid from stage III/IV HGSOC patients were isolated as described^8^ and imaged to determine their size distribution [Fig. 1(a)]. Spheroids consisted of a minority of the cellular makeup of the ascites, with a mean of 18% of the objects in the cellular portion of the ascites being spheroids vs single cells [Fig. 1(b)]. While larger spheroids were observed (up to 1 mm), the median diameter was 55 *μ*m, which is much smaller than most protocols to generate spheroids [Fig. 1(c)]. Cell phenotypes were assessed through IHC staining for tumor cells (pan-cytokeratin), immune cells (CD45), and fibroblasts (FSP1). There was no appreciable staining of immune cells or fibroblasts, suggesting the majority of spheroids consisted of tumor cells alone (supplementary material Fig. S1). As there were multiple cells in several spheroids that did not stain positively for pan-cytokeratin, we stained spheroids in ascites with PAX8, an additional epithelial tumor marker, and vimentin, a mesenchymal marker. Once again, our results suggested that spheroids were composed of only tumor cells, as nearly all cells in spheroids were positive for PAX8 and negative for vimentin (supplementary material Fig. S2). It is possible that mesenchymal or immune cells were attached to the exterior of spheroids and lost during isolation; if this is the case, the core of the spheroid is composed of tumor cells. Immunohistochemical (IHC) staining revealed that spheroids from ascites expressed diverse ECM proteins, including collagen I, fibronectin, collagen IV, and laminin [Figs. 1(d)–1(f), supplementary material Fig. S3).

**FIG. 2.**
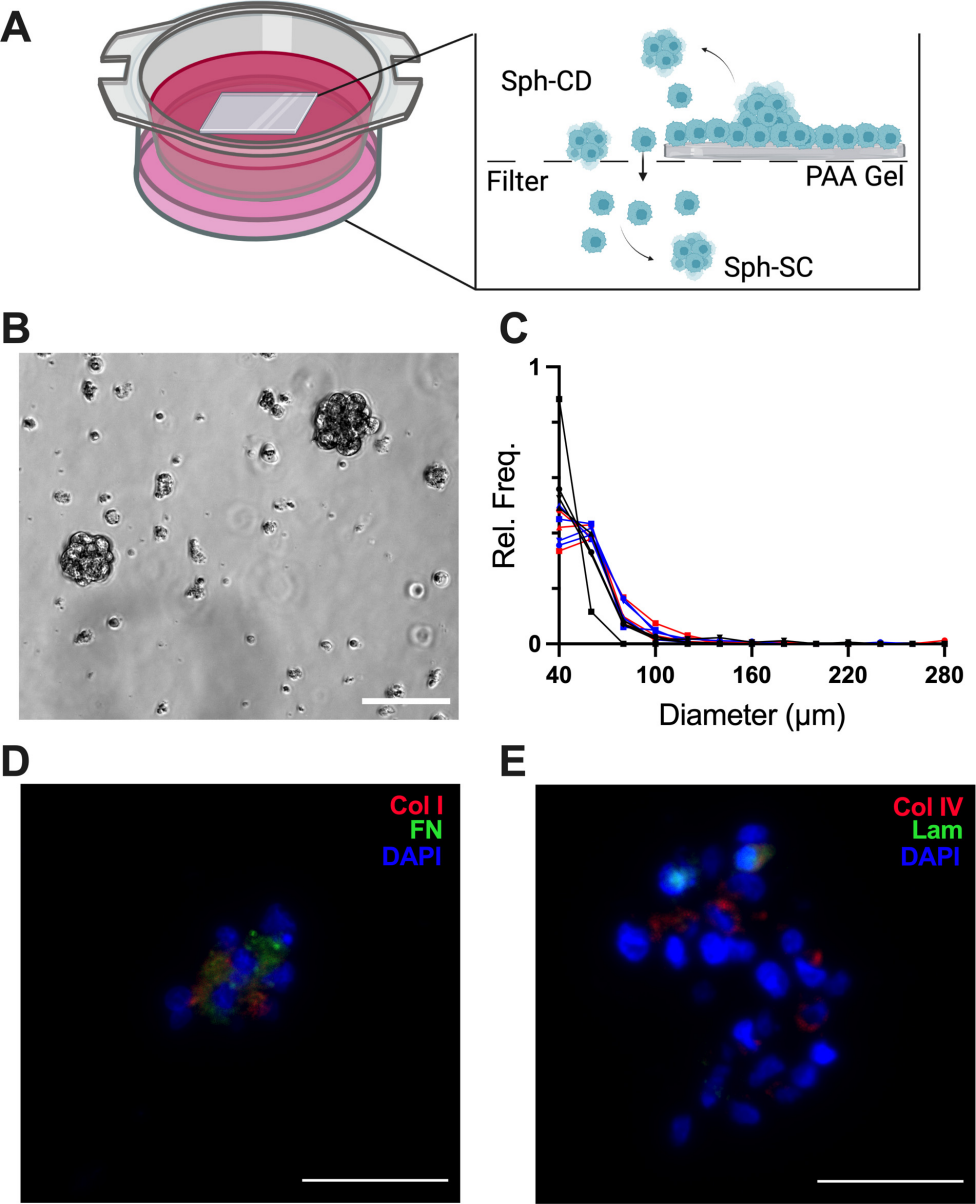
A novel *in vitro* model to isolate spheroids produced through collective detachment vs aggregation of detached single cells. (a) Schematic of *in vitro* model to study spheroid formation. Created with Biorender.com (b) phase images of Sph-CD isolated from the *in vitro* model. Scale bar = 100 *μ*m. (c) Histogram of the size distribution of Sph-CD isolated from the *in vitro* model. Shown are 11 *in vitro* distributions, each representing an experiment from OV90 (n = 4, black), OVCAR3 (n = 4, blue), and OVCAR8 (n = 3, red). (d)–(e) Immunohistochemistry of *in vitro* spheroids. (d) Collagen I (red), fibronectin (green), nuclei (blue) and (e) collagen IV (red), laminin (green), and nuclei (blue). Scale bar = 50 *μ*m.

**FIG. 3.**
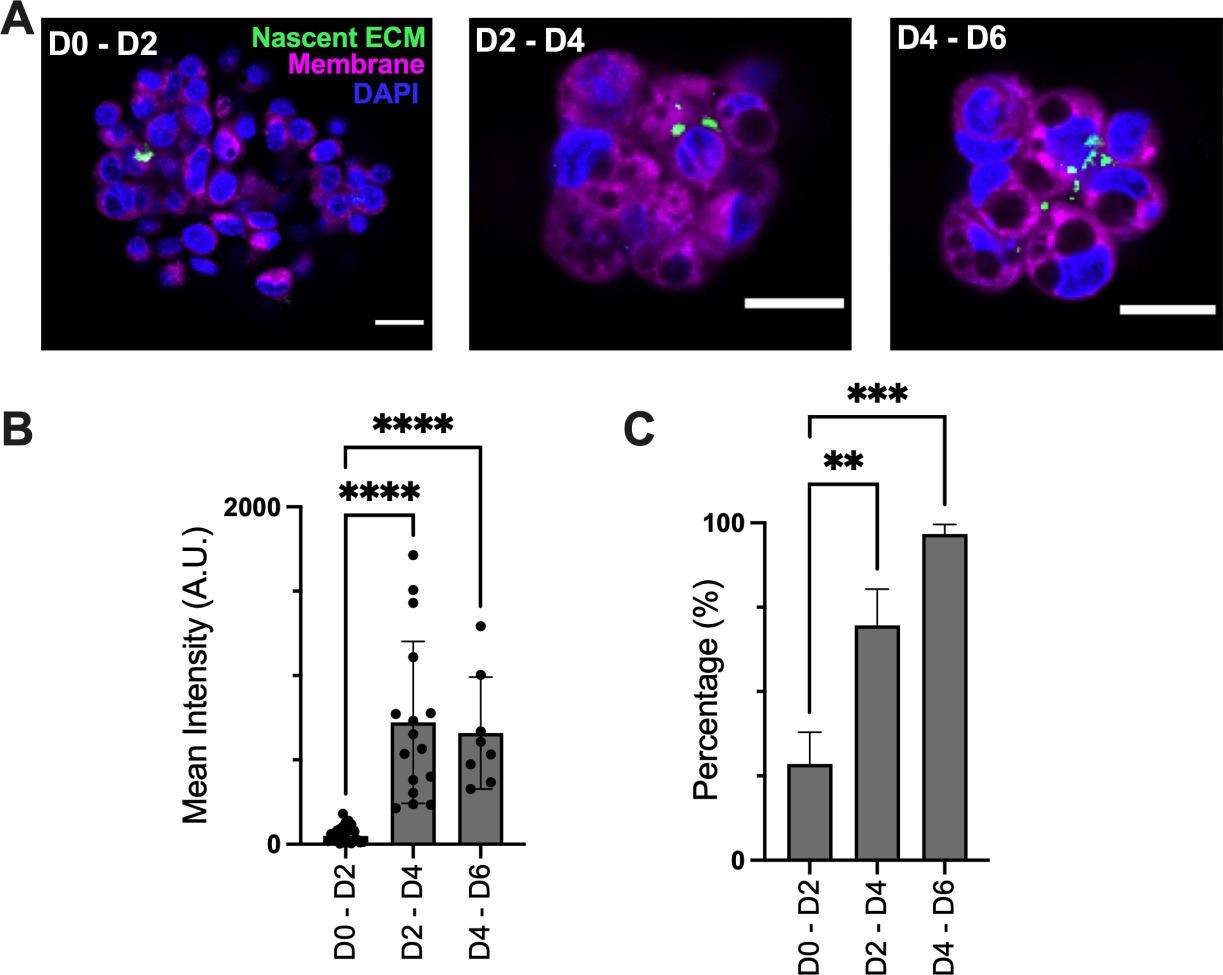
ECM was produced in spheroids after detachment. (a) Nascent ECM protein was visualized using AHA incorporation and DBCO-488 labeling. Spheroids were counterstained with Hoescht 33342 (1:1000) and CellMask Deep Red (1:1000), scale bar = 50 *μ*m. (b) Quantitative analysis of nascent signal across timepoints, n = 8–27 spheroids. ^****^ indicates p  <  0.0001 by one-way ANOVA with Dunnett's multiple comparisons. (c) Percentage of spheroids that have nascent protein across timepoints, n = 3 wells. Timeframe of AHA inclusion in culture is noted as days 0–2 (D0−D2), days 2–4 (D2−D4), and days 4–6 (D4 − 6) after detachment. ^*^ indicates p < 0.05 by one-way ANOVA with Dunnett's multiple comparisons.

#### Development of in vitro model and characterization of in vitro spheroids

Recent studies have shown that spheroids were produced not only through aggregation of exfoliated single cells but also through collective detachment of cells as a single unit.^6^ However, these studies did not isolate Sph-CD from Sph-SC. To separate Sph-CD from single cells that detach, we chose to culture cells on a coverslip on top of a 40 *μ*m cell strainer. Based on the median spheroid size, Sph-CD will get caught in the filter, whereas single cells that exfoliate are able to pass through the filter, after which they may aggregate together or remain as single cells [Fig. 2(a)]. Next, we sought to use a substrate that allowed for the tunability of different microenvironment factors relevant to the HGSOC tumor microenvironment, such as substrate stiffness and ECM identity and concentration. Polyacrylamide (PAA) gels allow for easy tailoring of substrate stiffness and ECM identity.^9^ For this study, the PAA gel was functionalized with collagen I, as collagen I is a major ECM component of both the ovary and the omentum.^10,11^ As most spheroids from ascites were composed of only tumor cells (supplementary material Fig. S1), we examined HGSOC tumor cell monolayers in our model.

When OV90, OVCAR3, or OVCAR8 cells were cultured in the model, spheroids were observed in both the supernatant/filter and the lower chamber of the well. To validate that the model was enriched for Sph-CD in the supernatant/filter vs Sph-SC in the lower chamber, cells were stained with either CellTracker Green or CellTracker Red and seeded on separate PAA gels. These gels were placed on top of the same filter, and the composition of the spheroids was assessed [supplementary material Fig. S4(a)]. In this setup, spheroids that contained both red and green-stained cells must originate from single cells from two different gels that aggregated after exfoliation. Spheroids found in the bottom chamber of the system had a significantly higher percentage of dual-colored spheroids, whereas spheroids found in the filter were primarily (81%) composed of one color [supplementary material Figs. S4(b) and S4(c)]. Confocal and phase imaging demonstrated that the monolayer or cells was interrupted by multi-layer areas that appeared to be budding off, further supporting that spheroids could result from collective detachment [supplementary material Figs. S4(d) and S4(e)]. Comparison of the number of Sph-CD, Sph-SC, and exfoliated single cells indicated that collective detachment was a rarer event (supplementary material Fig. S5).

**FIG. 4.**
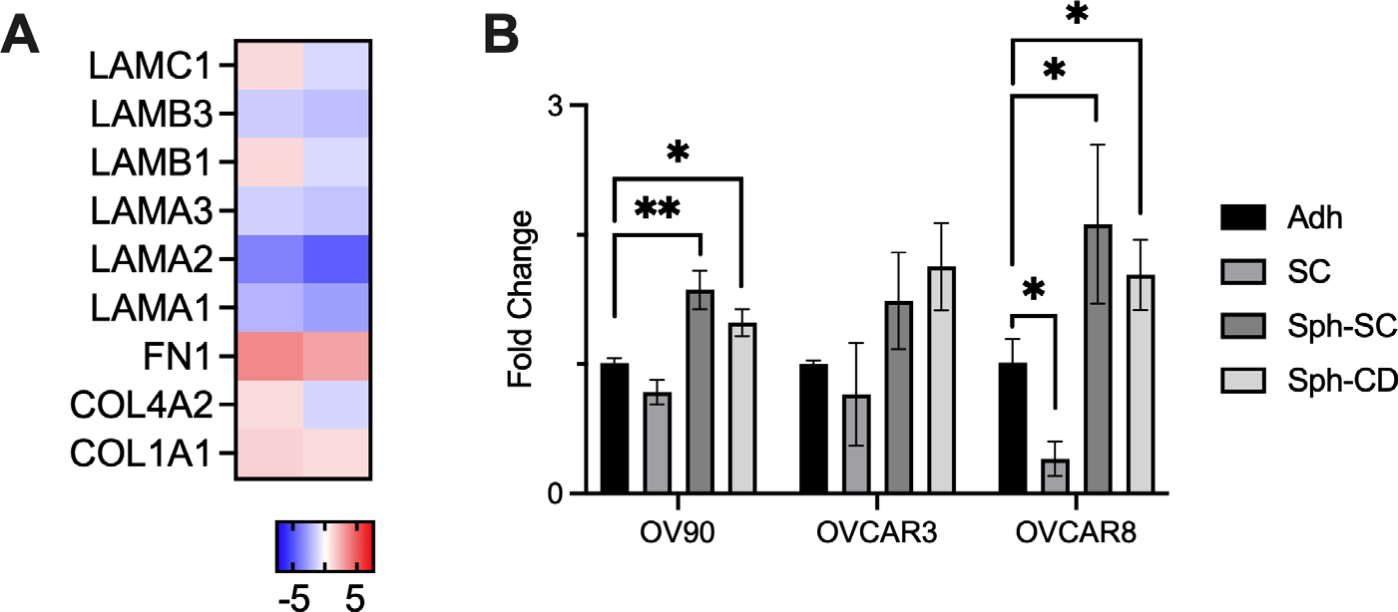
Fibronectin (*FN1*) is upregulated in HGSOC spheroids. (a) Fold change gene expression of selected ECM components for spheroids compared to single cells isolated from ascites, n = 2 matched patients. **(**b) Fold change of *FN1* expression between detached single cells (SC), Sph-SC, or Sph-CD, normalized to expression of cells remaining on the substrate (Adh), n  =  3 wells per condition. ^*^ indicates p < 0.05 by one-way ANOVA with Dunnett's multiple comparison relative to substrate.

**FIG. 5.**
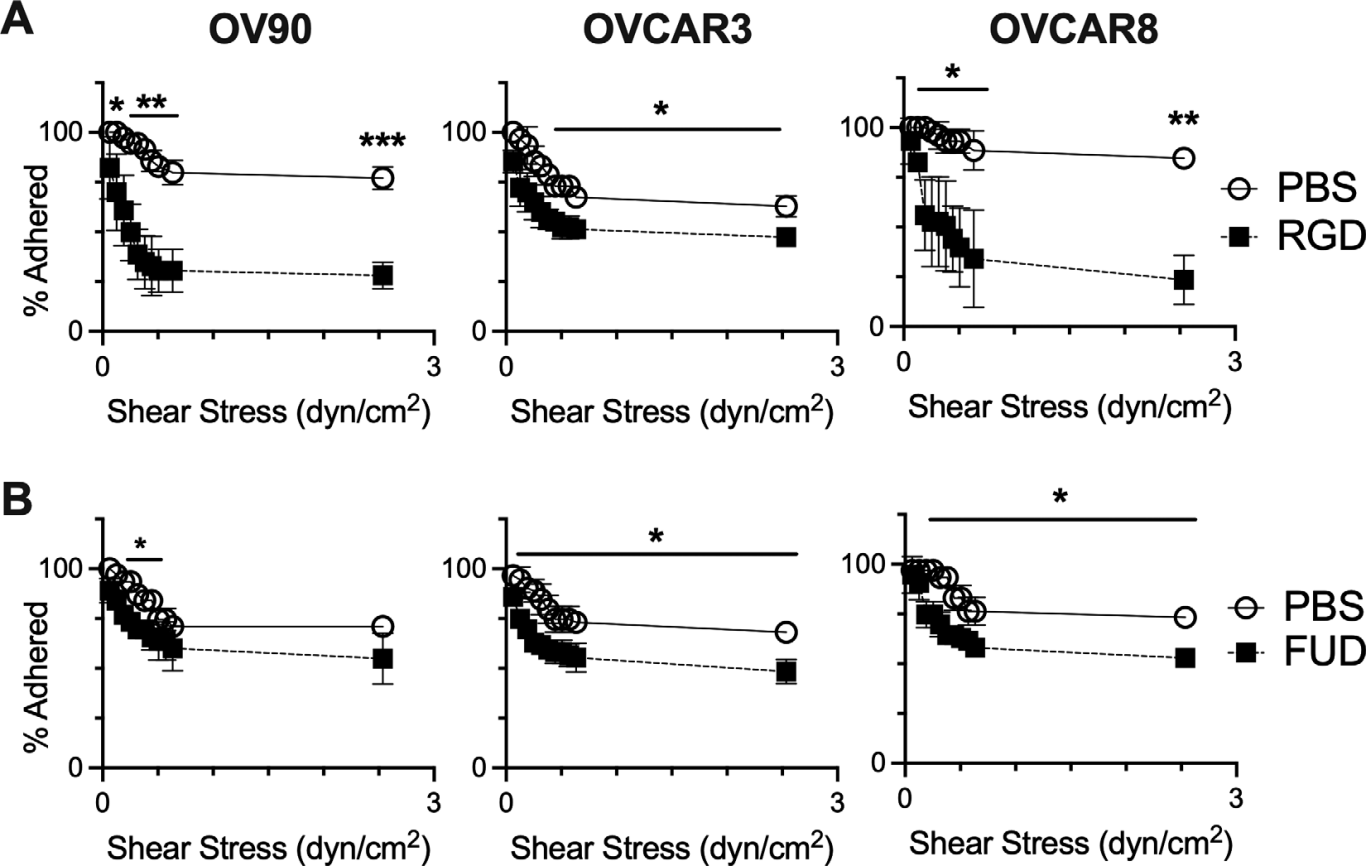
ECM in Sph-CD enhanced adhesion to mesothelial cells. (a) Percentage of spheroids that remained adhered to RGD- or vehicle-treated mesothelial cells as shear stress increased. (b) Percentage of PEG-FUD-treated or vehicle-treated spheroids that remained adhered to mesothelial cells as shear stress increased. n  = 3 chambers per condition, at least 20 spheroids per chamber. ^*^ indicates p  < 0.05, ^**^ indicates p < 0.01, ^***^ indicates p < 0.001 by unpaired t-test between trials at the same shear stress.

Sph-CD produced in this model had a similar morphology to spheroids from ascites [Fig. 2(b)]. Additionally, Sph-CD had a comparable size distribution to patient spheroids, with a median diameter of 51 *μ*m [p > 0.05 compared to patient spheroids, Fig. 2(c)]. To confirm that spheroids produced in the model were viable, calcein-AM and ethidium homodimer-1 staining was performed on Sph-CD, Sph-SC, and single cells. The staining demonstrated that cells in both Sph-CD and Sph-SC were viable up to 72 h after detachment [supplementary material Fig. S6(a)]. Additionally, spheroids exhibited a significantly higher percentage of live cells compared to single cells [supplementary material Fig. S6(b)]. We next compared viability between Sph-CD and Sph-SC. We determined that Sph-CD had significantly less caspase activity per cell compared to Sph-SC (supplementary material Fig. S7), indicating that Sph-SC have greater apoptotic activity. When *in vitro* Sph-CD were stained for ECM proteins, they displayed a similar expression profile as spheroids from ascites [Figs. 2(d) and 2(e), supplementary material Fig. S8], with expression of collagen I, fibronectin, laminin, and collagen IV.

### ECM is produced by tumor cells in spheroids after detachment

To test the hypothesis that cells took ECM deposited on the substrate with them, cells were seeded on hydrogels composed of labeled collagen, and the spheroids that detached from these gels were analyzed for associated fluorescent collagen I. Only 10% of spheroids had fluorescent collagen associated with the spheroid (supplementary material Fig. S9), suggesting that most of the ECM observed in Sph-CD was not taken from the surrounding environment during detachment. An alternative is that the ECM found in Sph-CD was produced by the tumor cells after they detached. To test this hypothesis, Sph-CD produced in the model were incubated with azide-containing methionine analog L-azidohomoalanine (AHA) to visualize newly synthesized extracellular proteins.^12^ As methionine is a component of most large proteins, this label does not indicate the presence of a particular protein, rather it indicates that ECM is being produced in general. AHA was incorporated into spheroid medium for two-day pulses across six days. Nascent ECM was detected at low levels during the first two days in suspension but was more strongly expressed during the following four days [Figs. 3(a) and 3(b)]. As there was variability in expression, we also quantified the number of Sph-CD with detectable nascent ECM, seeing an increase from less than 20% of spheroids during the first two days in suspension to nearly 100% during days four to six [Fig. 3(c)], suggesting that spheroids produced ECM after detaching.

### Fibronectin is upregulated in spheroids

As we observed that ECM was synthesized after detachment, we hypothesized that this may result from transcriptional changes in the Sph-CD. We first examined if there were differences in ECM expression between spheroids and single cells using paired samples from ascites [Fig. 4(a)]. Of the differentially expressed genes, *FN1* was upregulated in spheroids of both patients and is of particular interest as fibronectin has been previously shown to be highly upregulated with HGSOC progression,^13^ linked to metastatic processes such as migration and invasion,^14^ and associated with worse prognosis.^15^ We evaluated *FN1* expression between cells adhered to the substrate in the *in vitro* model, single cells that had detached, Sph-SC, and Sph-CD. *FN1* was upregulated in both Sph-CD and Sph-SC compared to the substrate cells, while cells that detached and remained as single cells had decreased *FN1* expression [Fig. 4(b)].

### Fibronectin in spheroids enhances adhesion to mesothelial cells

As we saw that fibronectin was abundant in spheroids from ascites and upregulated in Sph-CD, we next examined whether fibronectin could promote metastasis by enhancing adhesion of spheroids to mesothelial cells. To investigate this hypothesis, we employed a co-culture microfluidic system where Sph-CD flow along mesothelial cells to mimic being suspended in the peritoneal cavity. LP9 mesothelial cells were seeded into Ibidi microchannels, and CellTracker Green-labeled Sph-CD were added and allowed to adhere. After one hour, shear stress was increased by increasing the flow rate, and adhesion strength measured indirectly by counting the number of spheroids that remained adhered at a given shear stress. To determine the influence of fibronectin in Sph-CD on adhesion to LP9s, LP9s cells were pretreated with either soluble RGD or a vehicle control prior to adding the Sph-CD. RGD is the main adhesive peptide found in fibronectin^16^ and can block cell adhesion through competition for integrin binding.^17,18^ When LP9 cells were pretreated with RGD, spheroid adhesion was significantly weaker [Fig. 5(a)]. These results suggest that integrins on mesothelial cells interact with RGD-containing motifs of the Sph-CD ECM to strengthen adhesion. However, RGD is also found in other ECM proteins; therefore, to test the role of fibronectin more specifically, we next targeted its assembly into the matrix.

Though cells bind directly to fibronectin through α_5_β_1_ or α_v_β_3_ integrin interactions, fibronectin molecules also form an assembled matrix with each other and other ECM molecules to provide structural support for adhesion.^19,20^ To inhibit this matrix assembly, we employed a PEGylated fibronectin-binding peptide FUD (PEG-FUD) in the above-described co-culture system. PEG-FUD has been shown to inhibit fibronectin fibrillogenesis by acting as a competitive inhibitor of the type I FN domains to block FN dimerization but does not interact with the fibronectin type III domain responsible for cellular interactions.^21,22^ Sph-CD were produced in the *in vitro* model and treated with 1 *μ*M of PEG-FUD or vehicle for 24 h in non-adherent plates. These spheroids were added to LP9 cells seeded in microchannels, and after a one-hour incubation, increasing shear stress was applied. Spheroids treated with PEG-FUD had a significant decrease in adhesion strength [Fig. 5(b)]. Notably, PEG-FUD did not seem to interfere with spheroid integrity. Together, our results suggest that fibronectin expressed in Sph-CD strengthened adhesion to mesothelial cells.

## DISCUSSION

Several studies have reported that collective detachment represents one mode of metastasis in ovarian cancer,^5–7^ and spheroids are believed to have advantages in the metastatic process due to increased anoikis resistance and chemoresistance.^2–4^ Despite the potential importance of collective detachment in ovarian cancer, it is difficult to study the mechanisms that govern this process due to a lack of appropriate model systems. The *in vitro* model introduced in this work presents a platform to study collective detachment and subsequent behavior of Sph-CD and Sph-SC. We demonstrate that spheroids generated *in vitro* can be enriched for Sph-CD and validate that the spheroids formed in this model mimic those found in patients.

To the best of our knowledge, this is the first study to systematically characterize the size of spheroids from ascites. Though a small fraction of spheroids were considerably larger (>200 *μ*m in diameter), the majority of spheroids were small (between 50 and 75 *μ*m in diameter). These data should be considered when designing experiments with HGSOC spheroids. For example, hanging drop spheroids have been used to study the development of chemoresistance, but the spheroids appear to be 3–4 times larger in diameter,^23^ although smaller spheroids can be generated from as few as 10 cells.^24^ Sph-CD produced using our model have a similar size distribution to patient samples. Many protocols use Matrigel to encourage formation of spheroids; while patient spheroids expressed the main components of Matrigel (laminin and collagen IV), we also found evidence of stromal proteins such as collagen I and fibronectin. Sph-CD expressed a similarly diverse panel of ECM and appear to synthesize them after collective detachment, suggesting that use of ECM to form spheroids may not be physiologically relevant.

We saw that the spheroids in ascites consisted primarily of cytokeratin-positive tumor cells. A recent study looking at the role of the epithelial-mesenchymal transition (EMT) in HGSOC spheroids^25^ found similar results, where it was observed that spheroids in ascites were wholly composed of tumor cells. Cells in spheroids expressed epithelial markers including PAX8 and EpCAM, but a subset of cells also expressed proteins associated with a mesenchymal phenotype, including αSMA and fibronectin. *In vitro,* it has been suggested that cells of either epithelial or mesenchymal lineage are responsible for detachment.^6,7^ The cell lines we used here (OVCAR3, OVCAR8, and OV90) express the epithelial marker E-cadherin,^6,26,27^ but we demonstrated that they produce fibronectin, implying a level of cellular plasticity. Using models like the one presented here, further study of the role of EMT in collective detachment will be possible.

We determined that Sph-SC had higher apoptotic activity compared to Sph-CD activity as measured by caspase activity. These results suggest that anoikis-induced apoptotic cascades begin shortly after detachment from ECM and continue to impact cells even after they have aggregated. Additionally, this work proposes that collective detachment, and subsequent ECM production in spheroids, may be a route of achieving anoikis resistance in HGSOC metastasis. Indeed, other work has shown that overexpressing integrin α_v_β_3_ in HGSOC cells promoted anoikis resistance, in part, through increasing cell aggregation in non-adherent conditions,^3^ and that expression of ECM proteins such as versican is upregulated when HGSOC cell lines form spheroids.^28^

In order to form new tumors, metastasizing cells must attach to mesothelial cells that line peritoneal organs. Many prior studies^29^ have examined these interactions in static culture, but movements (e.g., walking, breathing, and digestion) set up flow and shear stress in the peritoneal microenvironment.^30^ Using the setup described here with individual tumor cells, we showed that interactions between CD24 on tumor cells and P-selectin expressed on mesothelial cells facilitates tethering and rolling under shear stress.^31^ Here, we demonstrate that fibronectin expressed in spheroids contributed to adhesion to mesothelial cells. Interestingly, it has been shown that integrins on isolated tumor cells bind to fibronectin expressed by mesothelial cells, facilitating adhesion.^32^ Together, these results suggest bi-directional ECM–integrin interactions exist to fortify adhesion in metastatic sites.

RGD was added to mesothelial cells prior to co-culture with Sph-CD to block mesothelial integrins from interacting with Sph-CD ECM, resulting in a decrease in adhesion strength. This suggests that fibronectin in spheroids is bound by integrins on the mesothelial cells, which include integrin α_5_, a sub-unit of the fibronectin receptor.^33^ In addition, our results pose that apart from possible direct fibronectin–integrin interactions, fibronectin matrix assembly strengthens spheroid adhesion to mesothelial cells. When fibronectin molecules assemble into fibrils, cryptic binding sites become exposed, allowing for the binding of growth factors, cytokines, and other ECM molecules.^34,35^ Interestingly, while fibronectin assembly has been shown to strengthen spheroid cohesion,^36^ treatment with PEG-FUD did not lead to significant changes in spheroid morphology, indicating that other ECM in the spheroid may support cohesion. Though the mechanism linking fibronectin fibrillogenesis and spheroid–mesothelial adhesion is still unclear, our data support that fibronectin fibrillogenesis impacted strength or stability of adhesions.

In this study, we present a novel *in vitro* model that can be used to produce Sph-CD that are similar in size as those found in patient ascites. Importantly, as the model is constructed from commercially available components and is simple to construct, it can easily be translated across labs. We show that Sph-CD produce their own ECM after detachment and that this matrix is similar in composition to the ECM of spheroids from ascites. Furthermore, spheroids have elevated *FN1* expression, which strengthens adhesion of Sph-CD to mesothelial cells. Together, this work provides a framework for studying the formation of HGSOC spheroids by collective detachment and evidence supporting a role for fibronectin in the dissemination of spheroids during metastasis.

## METHODS

Unless noted, all materials were purchased from ThermoFisher.

### Cell lines

All experiments used ovarian cancer tumor cell lines OV-90, OVCAR-3, and OVCAR-8 (ATCC). Cell lines were authenticated by human short tandem repeat (STR) analysis at the TRIP Lab at the University of Wisconsin–Madison. Cells were cultured in 1:1 Medium 199 (with Earle's salts and L-glutamine, Sigma-Aldrich) and MCDB 105 medium (Sigma-Aldrich). Experiments were conducted in serum-free medium, and maintenance culture included 15% heat-inactivated fetal bovine serum (FBS). LP-9 mesothelial cells (Coriell) were cultured in 1:1 Medium 199 and Ham's F-12 supplemented with 200 ng/mL hydrocortisone, 5 ng/mL epidermal growth factor, 0.75 mM L-glutamine, and 10% FBS. All media included 1% penicillin–streptomycin.

### Sample procurement

Ascites samples from Stage III/IV HGSOC patients were collected during debulking surgeries. The University of Wisconsin Carbone Cancer Center Translational Science BioCore acted as an honest broker under IRB #2016–0934, obtaining informed consent from all participants and de-identifying samples. The cellular portion was isolated from the fluid portion of the ascites by centrifuging at 300 g for 5 min. The fluid was removed and cells were resuspended in FBS. Single cells and spheroids were separated as previously described.^8^ Age, race, and staging information for samples used in this study can be found in supplementary material Table S1; all subjects were female as this is a gynecological cancer.

### Immunohistochemistry

Spheroids were isolated from single cells using a 40 *μ*M cell strainer, fixed for 15 min in 4% paraformaldehyde in PBS, and embedded in 1.5% agarose in water.^8^ The agarose plug was paraffin-embedded and 5 *μ*m sections were mounted onto slides for staining.

Slides were deparaffinized using sequential ethanol washes. Antigen retrieval was performed by incubating slides in citrate buffer (Vector Laboratories) for one hour at 80 °C. Slides were blocked overnight in normal horse serum at 4 °C. Primary antibodies were diluted in blocking buffer, applied to slides, and incubated overnight at 4 °C. Primary antibodies used included CD45 (R&D Systems MAB1430, 10 *μ*g/ml), FSP1 (Sigma-Aldrich 07–2274, 10 *μ*g/ml), collagen I (Abcam ab34710, 1:500), collagen IV (Abcam ab6311, 1:200), laminin (Abcam ab11575, 1:400), fibronectin (Abcam ab6328, 1:200), PAX8 (PA1–112, 1:300), vimentin (Sigma SAB4200716, 1:500), and pan-cytokeratin (Origene BP5069, 1:500). Secondary antibodies were diluted in blocking buffer, applied to slides, and incubated for one hour at room temperature protected from light. Secondary antibodies included Alexa Fluor 488 goat anti-mouse (1:500), Alexa Fluor 594 goat anti-rabbit (1:500), and Alexa Fluor 647 goat anti-guinea pig (1:500).

Slides were mounted using ProLong Diamond Antifade with DAPI and imaged on a Zeiss Axio Observer.Z1 inverted microscope with an AxioCam 506 mono camera with a Plan-Apochromat 20X 0.8-NA air objective.

### *In vitro* model

Polyacrylamide (PAA) gels were composed of 10% acrylamide (BioRad) and 0.45% bis-acrylamide (BioRad) and 0.5% Irgacure-2959 as a photoinitiator (Advanced Biomatrix). Silanized glass coverslips (Electron Microscopy Sciences) of 9 × 9 mm were placed on top of 19 *μ*l drops of prepolymer solution and cross-linked under UV light at 254 nm for 15 min. PBS was added to the gels and swelled overnight. 0.5 mg/mL Sulfo-SANPAH dissolved in 50 mM HEPES (pH 8.0) was added to gels, and UV light was applied for 25 minutes. Gels were washed 2× in 50 mM HEPES and 2× in PBS on a shaker. Then, 100 *μ*g/ml PureCol (Advanced Biomatrix) diluted in PBS was added to the gels and incubated overnight. The collagen solution was aspirated and 50 mM Tris-HCl was added to the gels and incubated at room temperature for 15 min to quench any remaining sulfo-SANPAH reactive groups. Gels were washed 3× with PBS, and UV light was applied for 30 min to sterilize.

OV90, OVCAR3, or OVCAR8 cells were seeded on gels at a density of 926 000 cells/cm^2^ in media with 15% serum. After four hours, the media was aspirated to remove any non-adherent cells and cells were washed one time with serum free medium. Using sterile tweezers, the coverslips were carefully placed in a 40 *μ*m cell strainer (Sigma) sitting in a six-well plate filled with 10 ml of serum free medium; 72 h was given for spontaneous detachment to occur. To collect the Sph-CD spheroids, the gels were removed from the filter, and the filter was carefully inverted on top of a 50 ml conical tube, and 4 ml of serum free medium was passed through the filter to bring the spheroids into the tube. The Sph-SC were collected by filtering the medium in the well through a separate 40 *μ*m cell strainer, and in the same manner, the Sph-CD spheroids were also collected. For some experiments, single cells that had detached but not aggregated were collected from the media that had passed through the strainer.

### Model validation

To test for viability, single cells and spheroids were collected from the model after 72 h, stained with 10 *μ*M calcein-AM and 5 *μ*M ethidium homodimer-1 for 30 min in serum free medium, and imaged. For validation of Sph-CD isolation, cells were stained with either 5 *μ*M CellTracker Green CMFDA or 5 *μ*M CellTracker Red CMTPX for 30 min in serum free medium prior to seeding on the PAA gels. For these experiments, one coverslip seeded with CellTracker Green-stained cells and one coverslip seeded with CellTracker Red-stained cells were placed in the same filter. After 72 h, spheroids were collected from both in and through the filter and imaged.

### Nascent ECM labeling

Sph-CD were collected at the end of the 72-h detachment period and placed in plates coated with 50 *μ*g/ml poly(2-hydroxyethyl methacrylate) (polyHEMA) to prevent spheroid attachment to the bottom of the plate. Newly synthesized ECM was labeled by incorporating 0.1 mM L-azidohomoalanine (AHA, Click Chemistry Tools), a methionine analog, into the culture medium. AHA was added, such that three separate time groups received AHA for two day pulses over a period of six days. DMEM without methionine was used in these experiments to prevent competition with AHA.

After the two day incubation with AHA, nascent ECM was visualized using fluorescent probe AZDye 488 DBCO (Click Chemistry Tools), which detects azide-tagged biomolecules. Spheroids were stained with 3 *μ*M AZDye 488 DBCO, 5 *μ*M ethidium homodimer-1 (to assess cell viability), 10 *μ*g/ml Hoechst 33342, and 5 *μ*g/ml CellMask DeepRed Plasma Membrane Stain for 30 min at 37 °C. Spheroids were washed twice with PBS and fixed for 15 min in 4% PFA in PBS prior to imaging. As the cells were not permeabilized, any nascent protein detected was extracellular. Images were taken on a Nikon A1RS Confocal Microscope with a 20× 0.75-NA air objective.

### Fluorescent labeling of collagen

To fluorescently label collagen, rat tail collagen (Advanced Biomatrix) was precipitated using 1 M NaCl. The collagen was redissolved in 2 M HCl and neutralized at a 4:1 volume of neutralization buffer (0.5 M NaCl and 0.1 M NaHCO_3_, pH 8.2). Alexa Fluor 488 5-TFP was added to the neutralized collagen and reacted for 1.5 h at room temperature on a shaker. The reaction was stopped by adding a 1:1 volume of stop buffer (1 mM glacial acetic acid and 1.5 M NaCl). Dialysis was performed on tagged collagen using the 10 K MWCO Slide-A-Lyzer Casettes with 0.02 N acetic acid. Dialysis was performed overnight, with the acetic acid exchanged three times. The labeled collagen was extracted from the dialysis cassette using a 22G needle, snap frozen in liquid nitrogen, and stored at −80 °C.

A quantity of 5 mg/ml collagen I gels were made with a 1:5 fluorescent collagen I incorporation. The collagen was neutralized with NaOH and polymerized for 1 h at 37 °C. Cells were seeded onto the gels at a concentration of 500 000 cells/cm^2^ overnight. Medium was changed to serum free medium, and spheroids were collected from gels after 72 h. Spheroids were imaged and analyzed for fluorescent collagen incorporation.

### Gene expression analysis

RNA from single cells and spheroids was isolated using the Monarch Total RNA Miniprep Kit (New England Biolabs), and cDNA was generated from this RNA using the SuperScript III First-Strand Synthesis System. An RT^2^ Profiler Array (Qiagen) designed for ECM and cell adhesion molecules was used to characterize gene expression with *ACTB*, *B2M*, *GAPDH*, *HPRT1*, and *RPLP0* as housekeeping genes. Fold regulation in gene expression was calculated using the ΔΔC_T_ method for spheroids relative to patient-matched single cells.

RNA isolation and cDNA synthesis from cells adhered to the substrate, detached single cells, Sph-CD, and Sph-SC was done as above. qRT-PCR was performed using 10× QuantiTect Primer Assay Kit for *FN1* (Qiagen) and SsoAdvanced Universal SYBR Green Supermix (Bio-Rad). *GAPDH* was used as a housekeeping gene, and fold change in *FN1* expression was calculated using the ΔΔC_T_ method relative to cells adhered to the substrate.

### Spheroid adhesion

*μ*-Slide VI 0.4 microchannels (Ibidi) were coated with 100 *μ*g/ml collagen I overnight. LP9 cells were seeded in microchannels at a density of 93 500 cells/cm^2^ in 30 *μ*l of LP9 medium as described.^31^ After 4 h, 60 *μ*l of additional medium was added to prevent evaporation and cells were incubated overnight. For the RGD experiments, the medium in the microchannels was changed to serum free medium and channels were treated with 20 *μ*M of soluble RGD peptide (Sigma-Aldrich) or PBS as a vehicle control for 3 h. For the PEG-FUD experiments, Sph-CD were collected and treated with 1 *μ*M of PEGylated recombinant FUD (PEG-FUD) (MW 27 390 Da) for 24 h in non-adherent plates.^21,37,38^ Approximately 1000 Sph-CD (collected from 12 devices) were added to each microchannel and incubated at 37 °C for one hour; this resulted in at least 25 spheroids in the field of view. Flow of serum free medium was applied to the microchannels for 30 s at a range of shear stresses (0.063–2.54 dyn/cm^2^), and videos were captured on a Zeiss Microscope at 5× magnification.

### Image analysis

Image analysis was done in FIJI.^39^ Phase images of the ascites cellular fraction were analyzed for object size using the “analyze particles” function to determine the percentage of “objects” in ascites that were spheroids vs single cells. Objects that had a diameter greater than 40 *μ*m were categorized as spheroids and objects that had a diameter between 15 and 25* μ*m were considered single cells. To quantify IHC staining and nascent protein signal of spheroids, a region of interest was created around the perimeter of the spheroids using the pan-CK signal. Signal was thresholded against the no primary control, and the mean intensity for each ECM channel was measured. Residual signal in the no primary sample after thresholding was then subtracted as background. For the nascent protein signal quantification, a spheroid with a mean gray value above 250 was considered to have nascent ECM expression.

### Statistical analysis

Groups were compared by an unpaired t-test or one-way ANOVA with a Dunnett's post-test. Statistical tests were performed in GraphPad Prism, p < 0.05 was considered significant.

## SUPPLEMENTARY MATERIAL

See the supplementary material for a table with patient demographics and nine additional figures.

## Data Availability

The data that support the findings of this study are available from the corresponding author upon reasonable request.
